# The Efficacy of Multidisciplinary Team Co-Management Program for Elderly Patients With Intertrochanteric Fractures: A Retrospective Study

**DOI:** 10.3389/fsurg.2021.816763

**Published:** 2022-02-24

**Authors:** Jixing Fan, Yang Lv, Xiangyu Xu, Fang Zhou, Zhishan Zhang, Yun Tian, Hongquan Ji, Yan Guo, Zhongwei Yang, Guojin Hou

**Affiliations:** ^1^Department of Orthopedics, Peking University Third Hospital, Beijing, China; ^2^Engineering Research Center of Bone and Joint Precision Medicine, Ministry of Education, Beijing, China

**Keywords:** intertrochanteric fractures, multidisciplinary team, traditional orthopedic care, China, elderly

## Abstract

**Background:**

Intertrochanteric fractures increased quickly in past decades owing to the increasing number of aging population. Recently, geriatric co-management was rapidly emerging as a favored clinical care model for older patients with hip fractures. The purpose of this study was to assess the efficacy of a multidisciplinary team (MDT) co-management program in elderly patients with intertrochanteric fractures.

**Methods:**

In this retrospective study, patients were divided into MDT group and traditional orthopedic care (TOC) group according to the healthcare model applied. 249 patients were included in the TOC group from January 2014 to December 2016 and 241 patients were included in the MDT group from January 2017 to December 2019. Baseline data, peri-operative data, and postoperative complications were collected and analyzed using SPSS 21.0.

**Results:**

No significant differences were observed between the two groups in terms of patient baseline characteristics. Patients in the MDT group had significantly lower time from admission to surgery and length-of-stay (LOS) compared with those in the TOC group. Furthermore, the proportion of patients receiving surgery within 24 h (61.4 *vs*. 34.9%, *p* < 0.001) and 48 h (80.9 *vs*. 63.5%, *p* < 0.001) after admission to the ward was significantly higher in the MDT group compared with those in the TOC group. In addition, patients in the MDT group had significantly lower proportion of postoperative complications (25.3 *vs*. 44.2%, *p* < 0.001), deep vein thrombosis (7.9 *vs*. 12.9%, *p* = 0.049), pneumonia (3.8 *vs*. 8.0%, *p* = 0.045) and delirium (4.1 *vs*. 9.2%, *p* = 0.025) compared with those in the TOC group. However, no significant changes were found for in-hospital and 30-day mortality.

**Conclusion:**

The MDT co-management could significantly shorten the time from admission to surgery, LOS, and reduce the postoperative complications for elderly patients with intertrochanteric fractures. Further research was needed to evaluate the impact of this model on patient health outcomes.

## Introduction

The incidence of hip fractures is increasing quickly in recent years owing to the longer life expectancy and increasing number of elderly patients (1). On a global scale, there were approximately 1.66 million hip fractures in 2000, and this number is expected to increase 6-fold by 2050, which would bring a heavy burden on the healthcare system (2). Early surgery is usually considered as a prior option for the treatment of hip fractures, which could enable early mobilization, good functional recovery, and fewer complications (3). The outcome might be extremely poor if there is prolonged bed rest. One study demonstrated that surgery within 48 h of admission could significantly reduce the mortality risk in hip fracture patients (4).

However, hip fractures generally occurred in elderly patients with multiple medical comorbidities, which could prolong the preoperative waiting time by assessing the medical comorbidities. Furthermore, older patients undergoing surgery had a higher risk of developing postoperative complications because of their underlying frailty profile (5, 6). Therefore, preoperative risk stratification and interventions tended to reduce postoperative complications and unplanned hospital readmissions in frail older patients (7). Recently, geriatric co-management has been rapidly emerging as a favored clinical care model for older patients with hip fractures, which could reduce the length-of-stay (LOS), mortality, postoperative complications, and unplanned hospital readmissions (8–10).

Intertrochanteric fractures accounted for 45% of hip fractures, while femoral neck fractures accounted for an additional 45% (11). It had been demonstrated that intertrochanteric fractures tended to have higher peri-operative hemoglobin (Hgb) drop than femoral neck fractures for anatomic reasons (12). Excessive blood loss had been shown to result in increased complications and a higher risk of perioperative death (13, 14). To the best of the author's knowledge, most of the previous studies accessing the efficacy of geriatric co-management consisted of both intertrochanteric fractures and femoral neck fractures. In addition, the application of orthogeriatric principles in clinical practice greatly varied depending on the healthcare system organization (15, 16). Currently, rare studies were available to investigate the efficacy of multidisciplinary team (MDT) co-management for elderly patients with intertrochanteric fractures in China. Therefore, the purpose of the present study was to evaluate the efficacy of the MDT co-management program on elderly patients with intertrochanteric fractures by comparing pre- and post-intervention outcomes in China.

## Materials and Methods

### Study Design

This was a pre-post retrospective study evaluating the outcomes of intertrochanteric fractures between the traditional orthopedic care (TOC) model and the MDT model at a level 1 trauma center. The patients in the TOC group were included from January 2014 to December 2017, and the patients in the MDT group were included from January 2017 to December 2019. Data were collected from the electronic medical records of all eligible patients. This study was approved by the institutional ethical review board of our institution (Peking University Third Hospital, Beijing, China).

The inclusion criteria were as follows: (1) acute intertrochanteric fractures (time from injury to admission was limited to 3 weeks); (2) age ≥60 years old; and (3) low-energy injury. A low-energy injury was defined as an injury which patients would sustain while falling over slippery ground in a walking or sitting position (17). The exclusion criteria were: pathological fracture; high-energy injury, such as car crash; peri-prosthetic fracture; multiply traumatized patients or terminal malignancies.

### Study Cohort

#### Multidisciplinary Team Model

The MDT model involved orthopedic surgeons, geriatricians, anesthesiologists, specialists of the intensive care unit (ICU), and physiotherapists. This model consisted of a pathway of care spanning Emergency Department (ED) presentation to discharge from hospital. Program implementation was led by an orthopedic trauma surgeon and coordinated by a geriatrician and an anesthesiologist. The traumatologist decided the suitability of the surgical treatment, technique to use, and when progressive weight-bearing could begin. The geriatrician managed comorbidities and polypharmacy to make patients clinically stable and ready for surgery, to reduce peri-operative complications and promote early functional recovery. A preoperative anesthesia evaluation was performed for risk assessment by an anesthesiologist within 12 h in the ED. Postoperative observation for 24–48 h in ICU was planned for high-risk patients. Furthermore, early geriatric rehabilitation was carried out by a physiotherapist. This team aimed to perform surgical treatment within 48 h upon admission and to achieve early discharge.

#### Traditional Orthopedic Care Model

When a patient suspected of having a fragility hip fracture was seen in the ED, an initial X-ray of the injured hip was obtained. Once the intertrochanteric fracture diagnosis was confirmed, the patient was admitted to the orthopedic ward under the care of an orthopedic surgical team. Treatment was generally managed by trauma surgeons and their team, who had no specific geriatric expertise. The geriatric assessment was not performed during inpatient treatment. Specialist consultants were called on according to their perception of patients' clinical conditions. The preoperative anesthesia assessment was performed on the day before surgery. Surgical treatment was performed by trauma surgeons. The patient would be transferred to the department of rehabilitation medicine after discharge.

### Data Collection

Data were retrospectively collected from the medical records by two independent researchers. Baseline data included patient demographic information [age, sex, bone mineral density (BMI), and injury side], hemoglobin value, comorbidities [such as hypertension, diabetes, dementia, coronary heart disease, and chronic obstructive pulmonary disease (COPD)], Charlson comorbidity index, type of surgery, American Society of Anaesthesiologists (ASA) classification, and fracture type according to the Arbeitsgemeinschaft für Osteosynthesefragen (AO). The following peri-operative data were retrieved: time-to-surgery from admission (days), blood transfusion, and LOS. Postoperative complications within 30 days were also collected: deep vein thrombosis (DVT), wound infection, pneumonia, urinary tract infection, cerebral vascular accident, acute coronary syndrome, gastrointestinal bleeding, postoperative delirium, in-hospital mortality, 30-day Harris score, and 30-day mortality. At the time of discharge, the patient was informed of a 1-month outpatient review. If the patient could not come to the clinic for review, we would contact the patient by telephone and assess the postoperative condition of the patient. A traumatologist would perform the outpatient review and assess the postoperative complications.

### Statistical Analysis

SPSS 21.0 software was used for statistical analysis (SPSS, Chicago, IL, USA). For quantitative data, the one-sample Kolmogorov–Smirnov test was used to test the normal distribution. Student's *t*-test or the Mann–Whitney test was used to compare continuous variables as appropriate. For qualitative data, the Chi-square test was used. Continuous variables were described as mean with *SD*, or in the case of non-parametric data as median with interquartile range. Categorical variables were described as numbers with corresponding percentages. A value of *p* < 0.05 was considered statistically significant, and all tests were two-sided.

## Results

### Baseline Characteristics

In the present study, a total of 513 intertrochanteric patients met the inclusion criteria. Of these 513 patients, 2 were pathological fractures, 11 were high-energy injury, 5 were peri-prosthetic fracture, and 5 patients were multiply traumatized patients. Overall, 23 patients were excluded from this study. Finally, 241 patients were included in the MDT group and 249 patients in the TOC group. Baseline characteristics for the MDT group and TOC group are summarized in Table 1. No significant differences were noted between the two groups in terms of patient baseline characteristics. Participants in the MDT group were slightly older than those in the TOC group (79.9 *vs*. 78.8, *p* = 0.053). Of these patients, 164 (68.1%) were female patients in the MDT group, and 173 (69.5%) were female patients in the TOC group. The average BMI was 22.7 (±4.3) kg/m^2^ and 23.4 (±4.4) kg/m^2^ in the MDT group and TOC group, respectively. With respect to the injury side, 136 (56.4%) had left side injury in the MDT group and 120 (48.2%) had left side injury in the TOC group. Patients belonging to the MDT group had a similar level of hemoglobin at admission compared with those in the TOC group (113.1 *vs*. 113.5, *p* = 0.823). In addition, similar levels of comorbidities were found in both the MDT group and TOC group, such as hypertension (59.8 *vs*. 58.6%, *p* = 0.802), diabetes (34.0 *vs*. 29.7%, *p* = 0.306), dementia (4.1 *vs*. 3.2%, *p* = 0.582), coronal heart disease (21.2 *vs*. 22.1%, *p* = 0.803), and COPD (11.6 *vs*. 9.2%, *p* = 0.388). The Charlson index score and the distribution of patients with Charlson index score >4 resembled the comorbidity distribution, and no differences were found between the two groups (2.3 *vs*. 2.2, *p* = 0.329 and 15.8 *vs*. 17.3%, *p* = 0.655, respectively). Furthermore, there were no significant differences between the two groups regarding ASA score, AO/OTA classification, and type of surgery.

**Table 1:** Baseline characteristics of the participants grouped by orthogeriatric co-management and orthopedic usual care.

**Characteristic**	**MDT group (*N* = 241)**	**TOC group (*N* = 249)**	***P*-value**
Age (mean years ± SD)	79.9 ± 8.1	78.8 ± 7.2	0.053
Women, *N* (%)	164 (68.05)	173 (69.48)	0.733
BMI (kg/m^2^)	22.7 ± 4.3	23.4 ± 4.4	0.059
Injury side (left/right)			0.068
Left, *N* (%)	136 (56.4)	120 (48.2)	
Right, *N* (%)	105 (43.6)	129 (51.8)	
Hgb at admission (g/L)	113.1 ± 18.6	113.5 ± 19.7	0.823
Hypertension, *N* (%)	144 (59.8)	146 (58.6)	0.802
Diabetes, *N* (%)	82 (34.0)	74 (29.7)	0.306
Dementia, *N* (%)	10 (4.1)	8 (3.2)	0.582
Coronary heart disease, *N* (%)	51 (21.2)	55 (22.1)	0.803
COPD, *N* (%)	28 (11.6)	23 (9.2)	0.388
Charlson comorbidity index, M ± SD	2.3 ± 1.3	2.2 ± 1.4	0.329
Charlson comorbidity index ≥4, *N* (%)	38 (15.8)	43 (17.3)	0.655
ASA class			0.403
1/2, *N* (%)	210 (87.1)	223 (89.6)	
3/4, *N* (%)	31 (12.9)	26 (10.4)	
AO/OTA classification			0.662
31-A1, *N* (%)	49 (20.2)	50 (19.8)	
31-A2, *N* (%)	162 (66.9)	176 (69.8)	
31-A3, *N* (%)	31 (12.8)	26 (10.3)	
Type of surgery			0.973
Intramedullary fixation, *N* (%)	223 (92.5)	229 (92.0)	
Extramedullary fixation, *N* (%)	8 (3.3)	9 (3.6)	
Other, *N* (%)	10 (4.1)	11 (4.4)	

*MDT, multidisciplinary team; Hgb, hemoglobin; TOC, traditional orthopedic care; BMI, bone mineral density; COPD, chronic obstructive pulmonary disease; ASA, American Society of Anaesthesiologists; AO, Arbeitsgemeinschaft für Osteosynthesefragen*.

### Clinical Indicators

**Figure 1:**
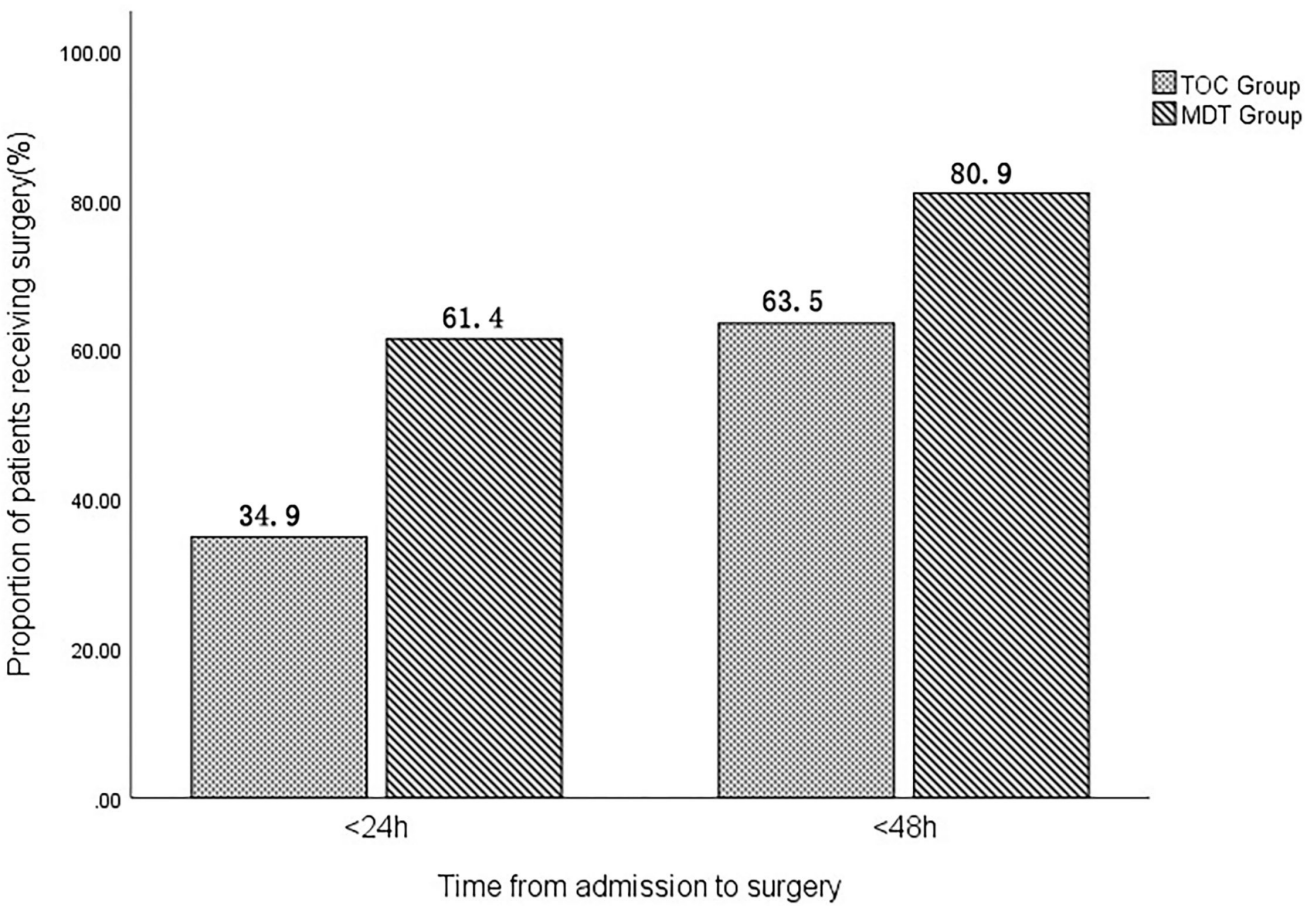
The proportion of patients receiving surgery within 24 and 48 h.

Clinical indicators grouped by the MDT and TOC models were presented in Table 2. The time-to-surgery from admission was significantly lower in the MDT group (1.7 ± 1.3 days) than in the TOC group (2.4 ± 1.5 days). Furthermore, the proportion of patients receiving surgery within 24 h (61.4 *vs*. 34.9%, *p* < 0.001) and 48 h (80.9 *vs*. 63.5%, *p* < 0.001) after admission to the ward was significantly higher in the MDT group compared with those in the TOC group (Figure 1). In addition, the total LOS was significantly lower in the MDT group (4.0 ± 2.5 days) than in the TOC group (5.0 ± 2.8 days).

**Table 2:** Clinical indicators grouped by orthogeriatric co-management and orthopedic usual care.

**Outcome**	**MDT group (*N* = 241)**	**TOC group (*N* = 249)**	***P*-value**
Time to surgery, days, M ± SD	1.7 ± 1.3	2.4 ± 1.5	<0.001
Time to surgery <48 h, *N* (%)	195 (80.9)	158 (63.5)	<0.001
Early surgery (<24 h), *N* (%)	148 (61.4)	87 (34.9)	<0.001
Length of stay, days, M ± SD	4.0 ± 2.5	5.0 ± 2.8	<0.001
RBC transfusion, *N* (%)	80 (33.2)	75 (30.1)	0.464
Hb >11 at discharge, *N* (%)	58 (24.1)	37 (14.9)	0.010
Postoperative Complications, *N* (%)	61 (25.3)	110 (44.2)	<0.001
DVT, *N* (%)	18 (7.9)	32 (12.9)	0.049
Wound infection, *N* (%)	3 (1.2)	4 (1.6)	0.736
Pneumonia, *N* (%)	9 (3.8)	20 (8.0)	0.045
UTI, *N* (%)	6 (2.5)	10 (4.0)	0.342
Delirium, *N* (%)	10 (4.1)	21 (9.2)	0.025
CVA, *N* (%)	5 (2.1)	6 (2.4)	0.802
ACS, *N* (%)	8 (3.3)	13 (5.2)	0.299
GI bleeding, *N* (%)	2 (0.8)	4 (1.6)	0.435
30-day Harris Score, M ± SD	80.8 ± 7.5	80.3 ± 7.0	0.440
In-hospital mortality, *N* (%)	1 (0.4)	2 (0.8)	0.582
30-day mortality, *N* (%)	4 (1.7)	6 (2.4)	0.557

*RBC, red blood cell; DVT, deep venous thrombosis; UTI, urinary tract infection; CVA, cerebral vascular accident; ACS, acute coronary syndrome; GI, gastrointestinal; RTI, respiratory tract infection; MI, myocardial infarction*.

The proportion of patients receiving RBC transfusion was similar between the MDT group and the TOC group (33.2 *vs*. 30.1%, *p* = 0.464). However, a higher percentage of patients in the MDT group had hemoglobin >11 g/dl compared with the TOC group at discharge (24.1 *vs*. 14.9%, *p* = 0.010). With regard to the postoperative complications, patients in the MDT group had significantly lower proportion of postoperative complications (25.3 *vs*. 44.2%, *p* < 0.001), DVT (7.9 *vs*. 12.9%, *p* = 0.049), pneumonia (3.8 *vs*. 8.0%, *p* = 0.045), and delirium (4.1 *vs*. 9.2%, *p* = 0.025) compared with those in the TOC group. However, no statistically significant differences were observed in wound infection, urinary tract infection, cerebral vascular accident, acute coronary syndrome, and gastrointestinal bleeding (*p* > 0.05). In addition, the average Harris score was 80.8 (±7.5) in the MDT group and 80.3 (±7.0) in the TOC group, and no significant difference was found between the two groups.

With regard to in-hospital and 30-day mortality, there was a reduction in mortality during the study period. We observed a lower in-hospital and 30-day mortality rating in the MDT group compared with the TOC group (0.4 *vs*. 0.8% and 1.7 *vs*. 2.4%, respectively), but no statistical significance was found (Table 2, *p* > 0.05).

## Discussion

Hip fractures were associated with significant morbidity, mortality, and loss of independence (18). Intertrochanteric fractures were one of the hip fractures, which commonly occurred in the elderly and caused a high mortality rate due to the loss of walking ability (19). Unlike femoral neck fractures, intertrochanteric fractures tended to have a higher preoperative hemoglobin (Hgb) drop for anatomic reasons, which would increase the postoperative complications and the risk of perioperative death (12, 14). To reduce mortality and disability rate, early operation was crucial for good functional outcome and the avoidance of serious postoperative complications (20, 21). However, the best perioperative care for intertrochanteric femoral fractures remained controversial.

Recently, geriatric co-management has been rapidly emerging as a favored clinical care model for older patients with hip fractures, which could improve the care pathway and reduce the time from admission to surgery (22). However, the efficacy of the geriatric co-management might be affected by fracture type and the healthcare system organization (15, 16). Currently, few studies had investigated the efficacy of MDT co-management for elderly patients with intertrochanteric fractures in China. In the present study, we found that the implementation of MDT co-management for elderly patients with an intertrochanteric fracture was associated with a reduced time-to-surgery, reduced LOS, and reduced postoperative complications. Nevertheless, neither the in-hospital nor the 30-day mortality rate was affected by the application of MDT co-management.

Time-to-surgery was the most investigated parameter in hip fracture patients due to its undisputed influence on postoperative complications and mortality. Surgical delay had been shown to be an important indicator of a higher risk of postoperative complications in several studies (23, 24). In the present study, time-to-surgery was significantly reduced after the implementation of the MDT co-management (1.7 *vs*. 2.4 days) for the treatment of intertrochanteric fractures. Time-to-surgery in this study was lower than that observed in previous studies (25, 26), which might be influenced by several modifiable system variables, such as the availability of theater, drug treatments, and weekday admission (27). Additionally, a significantly higher percentage of patients received the surgery within 48 h after the admission in the MDT group compared with those in the TOC group. Several studies had found that a surgical delay of more than 48 h increased the risk of death (4, 28). Furthermore, the proportion of patients receiving early surgery (<24 h) in the MDT group was significantly higher than patients in the TOC group. The possible reason for reduced time-to-surgery might be that close MDT collaboration could speed up the preoperative assessment. Similarly, another study showed that coordinated, region-wide efforts to improve the timeliness of hip fracture surgery could successfully reduce the time-to-surgery (29). Whether shorter the time-to-surgery positively affects clinical outcomes needs to be investigated in the future. We merely observed a correlation between shorter time-to-surgery and lower incidence of complications and shorter LOS in this study.

The length of the hospital stay for patients following hip fractures was often reported as an outcome measure. The previous study had demonstrated that the reduction in LOS by orthogeriatric care models could lead to an additional reduction in costs because hospital costs accounted for 44% of direct costs for hip fracture patients (30, 31). In the present study, the LOS decreased by an average of 1 day from 5 to 4 days (*p* < 0.001) as a result of the active involvement of MDT co-management. This was similar to the average LOS of 4.6 days reported by Friedman et al. (32), and much shorter than the average LOS of 12 days reported by Christiano et al. (33). This discrepancy was likely due to differences in the patient population. Additionally, a large national cohort demonstrated that the reduction in LOS did not coincide with an increase in readmissions which was encouraging (34). Intertrochanteric fracture patients who had received MDT co-management had better mobilization and subsequently were more likely to discharge earlier rather than require longer nursing care in hospital, and therefore resulting in a corresponding decrease in cost.

With respect to the postoperative complications, a postoperative complication rate up to 59% had been reported in elderly people with hip fractures (35, 36). In the present study, a comparable rate (44.2%) of postoperative complications was found in the TOC group, which was significantly higher than that in the MDT group (25.2%). Additionally, the most common postoperative complications were DVT, pneumonia, and delirium. The overall incidence of DVT, pneumonia, and delirium was 10.2, 5.9, and 6.3%, respectively. In addition, the incidence of DVT, pneumonia, and delirium was 7.9, 3.8, and 4.1% in the MDT group, which was significantly lower than that in the TOC group. The possible reason might be to shorten the time-to-surgery, which could enable early mobilization. Not only does early mobility decrease the risk of DVT, pneumonia, delirium, and other postoperative complications developing in older patients, but also had been shown to reduce the incidence of fragility fractures in geriatric populations (37). Furthermore, it had been reported that the greater peri-operative blood loss was poor prognosis factors of postoperative complications (i. e., pneumonia, urinary tract infections, and DVT), the length of hospital stay, readmission rate, physical performance, and functional recovery (11). In the present study, the proportion of patients with Hb > 11 g/L at discharge was significantly higher in the MDT group than that in the TOC group. This might also partially explain the lower rates of postoperative complications in the MDT group.

Considering the postoperative mortality, we observed a reduction in the number of deaths in patients under collaborative models in our study, but those differences did not demonstrate statistical significance. The reduction of the in-hospital and 30-day mortality rate could be due to different causes. The decrease in the number of patients receiving conservative treatment might be one of the influencing factors (38), and some studies considered that early surgical treatment was directly related to the mortality of hip fractures (39, 40). However, Kristensen et al. (41) described a reduction in mortality independent of the surgical delay in a comparative study of orthogeriatric and ordinary orthopedic units, and Lund et al. (42) reported that neither the surgical delay nor the duration of the intervention was statistically significant risk factors for mortality after hip fracture surgery.

Several limitations existed in the present study. First, this was a retrospective study, which could have introduced a bias, since clinical records were drafted by different physicians. Second, the patient population was relatively small and the follow-up period was somewhat short. Third, all patients in this study came from one trauma center. Therefore, a multi-center large sample study would be required to validate our findings.

## Conclusion

In the present study, we found a relevant improvement with the implementation of MDT co-management for the treatment of intertrochanteric fractures in elderly patients. As a result, patients in the MDT group had a shorter time-to-surgery, shorter LOS, and lower rates of postoperative complications when compared with those in the TOC group. Furthermore, our MDT co-management could significantly improve the overall proportion of patients receiving surgery within 24 and 48 h. Therefore, MDT co-management might be an alternative model for the treatment of intertrochanteric fractures in elderly patients. Further research was needed to strengthen the advantages of the MDT co-management of older adults with intertrochanteric fractures.

## Data Availability Statement

The raw data supporting the conclusions of this article will be made available by the authors, without undue reservation.

## Ethics Statement

The studies involving human participants were reviewed and approved by the institutional ethical review board of Peking University Third Hospital, Beijing, China. The patients/participants provided their written informed consent to participate in this study. Written informed consent was obtained from the individual(s) for the publication of any potentially identifiable images or data included in this article.

## Author Contributions

FZ designed the study. JF and YL were responsible for the data collection and analysis and wrote the manuscript. ZZ, HJ, YT, and YG were responsible for the data collection. ZY, GH, and XX were responsible for data analysis and interpretation. All authors read and approved the final manuscript.

## Funding

This study was supported by the National Key R&D Program of China (grant no. 2018YFF0301102), National Natural Science Foundation of China (No. 81702127), and Peking University Third Hospital (grant no. Y62419-06).

## Conflict of Interest

The authors declare that the research was conducted in the absence of any commercial or financial relationships that could be construed as a potential conflict of interest.

## Publisher's Note

All claims expressed in this article are solely those of the authors and do not necessarily represent those of their affiliated organizations, or those of the publisher, the editors and the reviewers. Any product that may be evaluated in this article, or claim that may be made by its manufacturer, is not guaranteed or endorsed by the publisher.
